# Technical note: Image guided internal fiducial placement for stereotactic radiosurgery (CyberKnife)

**DOI:** 10.4103/0971-3026.76043

**Published:** 2011

**Authors:** Govindarajan Janardan Mallarajapatna, Sridhar Papaiah Susheela, Kumar Gangadharaiah Kallur, Nagaraj Kanakapura Ramanna, Prashant Guthlu Ramachandra, Sudhakar, Shivakumar Swamy Shivalingappa

**Affiliations:** Department of Imaging and Interventions, Health Care Global - Bangalore Institute of Oncology, Bangalore, India

**Keywords:** Fiducials, interventional, stereotactic radiosurgery

## Abstract

Internal gold fiducials are necessary for tracking the translational and rotational movements of target lesions during stereotactic radiosurgery. The fiducials are generally placed under image guidance in and around the lesions by interventional radiologists. Specific challenges are encountered during the procedure. This article discusses the basic principles and techniques as well as the specific complications.

## Introduction

CyberKnife is the first FDA-approved device for full-body dynamic radiosurgery and was developed by Accuray in 2001.[1–3] It delivers non-isocentric, non-coplanar, megavoltage photon radiation beams to irregularly shaped targets by using bony structures (for the skull and spine) or implanted internal markers (for extracranial sites) as landmarks, and also provides sub-millimeter accuracy.[4,5] It can be used to treat tumors in any intra- or extracranial region that cannot be managed by surgery.[6–10]

Understandably, a mechanism is necessary to track the lesions’ translational and rotational movements so as to achieve high (sub-millimeter) accuracy and safety. Lesions can be tracked either using bones or internal fiducial markers.

**Figure 1:**
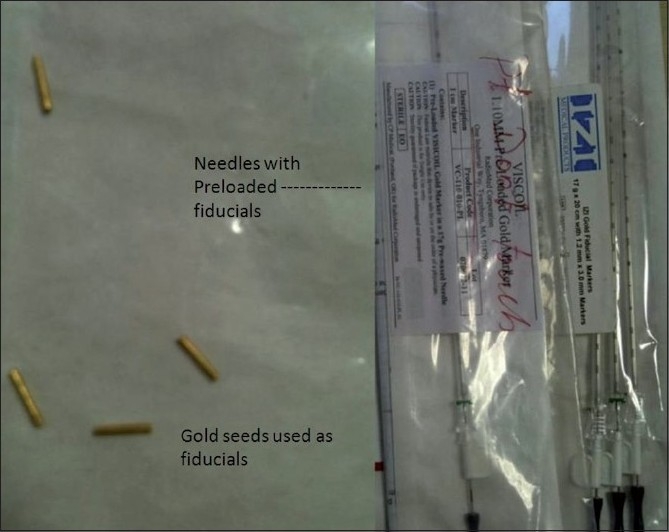
Photograph of free gold seeds and the introducers

The fiducials are internal markers, made of 99% gold and can be radiographically visualized. They are available [Figure 1] as 0.8 × 5 mm gold seeds in an unsterile pack or 17G needles preloaded with one 1.2 × 5 mm gold seed each. Visicoil (IBA Dosimetry America, Bartlett, USA) (0.75 mm diameter and 20 mm length) is a coiled gold fiducial; it prevents migration because of grooves on the surface but is relatively expensive. Fiducials can be placed through any route used for biopsy or fine-needle aspiration cytology. The percutaneous method is used for most of the thoracic and abdominopelvic masses. Transrectal, transurethral, or transperineal routes can be used for prostate lesions. Endoscopic/bronchoscopic and peroperative methods can be used when percutaneous methods are not possible.

With the patient on the treating table, two 45°- angled radiographs are obtained centering the beam at the lesion every few seconds. Identifying fiducials as distinct entities on these radiographs is necessary for tracking lesion movement during treatment and optimal conditions for this include a minimum of 2 cm distance between any two fiducials and a minimum of 15° degree angle within any group of three fiducials. To track rotational movement, a minimum of three fiducials placed in three orthogonal planes is necessary. Once in the body, there will be a small amount of migration of the fiducials by a few millimeters for the first few days but by one to two weeks, migration is assumed to be nil due to development of fibrosis around the fiducials. Also, if the fiducial is placed at the periphery of the lung or any other visceral organ, there is a possibility that it may move into the pleural and peritoneal cavities, respectively.

Though CT scan is a popular guiding tool for percutaneous fiducial placement for CyberKinfe treatment, there are no major studies regarding this. In a recent retrospective study of 105 consecutive patients, Sotiropoulou *et al*. have reported the successful use of CT scan for this purpose and have concluded that it is an accurate and safe method.[11] No major complications were reported in their study except for one patient who needed chest tube placement for pneumothorax.

## Technical Note

**Figure 2:**
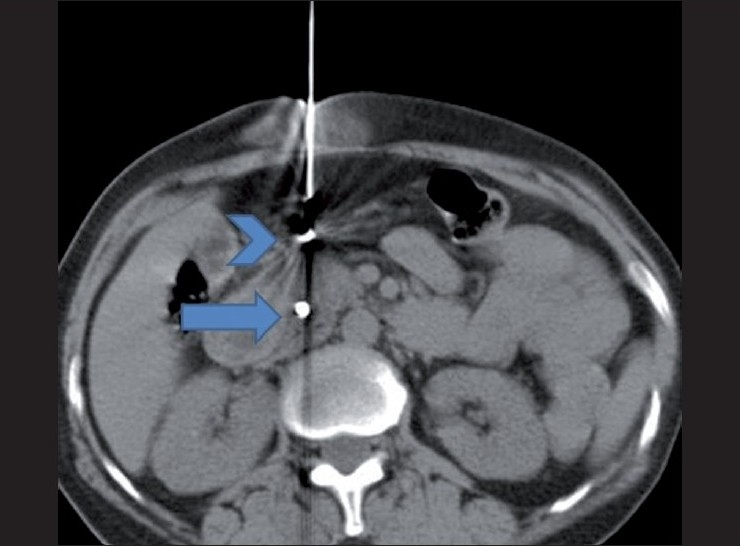
Axial CT scan after placement of a fiducial (arrow) in the pancreatic head and withdrawal of the needle (arrowhead) to change its direction

**Figure 3:**
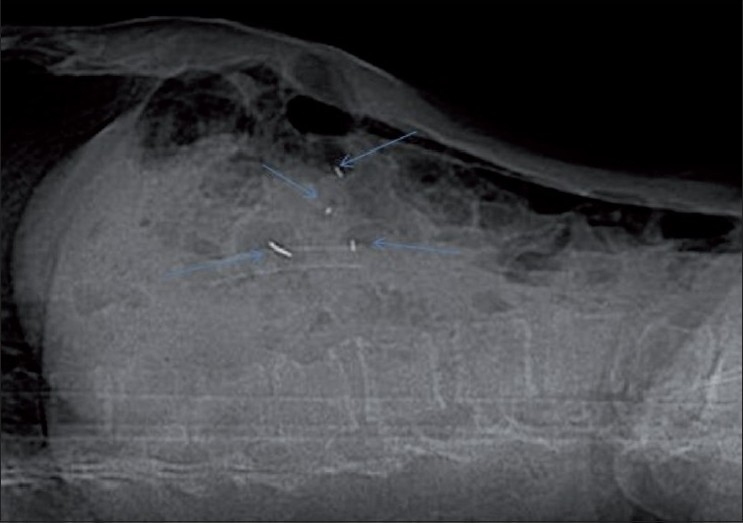
Lateral scanogram after placement of the fiducials under CT scan guidance, shows all the fiducials as well defined radiodense foci (arrows)

**Figure 4:**
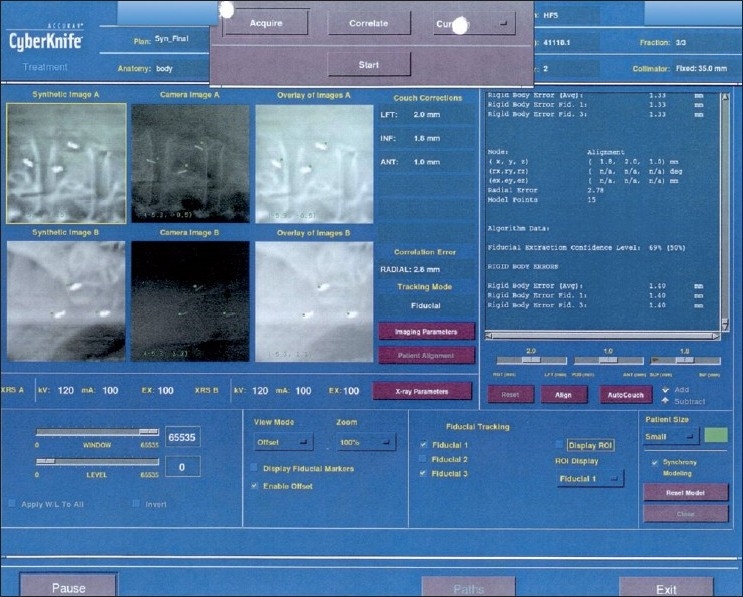
Images of the radiographs used to track the fiducials at the time of planning and execution of the CyberKnife treatment, demonstrate optimal positioning of the fiducials, which should form a triangle on 45°-angled radiographs

As in all percutaneous procedures, preparation starts with achieving a normal coagulation profile, ruling out active infection and obtaining informed consent. Three days of intravenous antibiotic (amikacin) along with oral ciprofloxacin and tinidazole for five days starting from day one are routinely given at our institute whenever small or large bowel transgression is contemplated. Under aseptic precautions and local anesthesia, an 18G Chiba or lumbar puncture needle is inserted under CT scan or USG guidance. The fiducial is advanced through the needle after confirming that the position of the needle tip is in or around the lesion. The final location of the needle tip is always confirmed by imaging to ensure that each fiducial is placed at a different site, within or near the lesion of interest. Confirmatory axial slices and topograms should be obtained [Figures 2–4]. Patients remain under surveillance for 2 h and are discharged with post-procedure instructions; they are asked to report back if they experience chest pain, breathlessness, fever, chills, or any unexpected symptoms.

A total of 122 patients have undergone image-guided fiducial placement in our department for the purpose of stereotactic radiosurgery for various malignant conditions in the last 11 months. Percutaneous (90 CT-guided, 5 USG-guided) and transrectal (27 TRUS-guided for prostate cancer) methods have been used and a total of about 450 gold fiducials have been placed. The target lesions were in the chest (25 lungs/pleura/chest wall and mediastinum), the abdomen (37 liver/gallbladder/common bile duct/pancreatic) and the pelvis (27 prostate, 33 gynecological).

## Results

Three or more fiducials were tracked in most of the patients. In a minority of patients, the fiducials migrated and were not tracked by the CyberKnife. Three patients had one fiducial each migrating into the pleural cavity, five patients had one fiducial each migrating into the peritoneum and 14 patients had intra-organ migration in the lung or viscera. Intra-organ migration generally does not result in the loss of a fiducial, and since no migration is expected within the organ after 7-14 days, planning of treatment is generally done 14 days after fiducial placement. One patient had a significant pneumothorax, for which, a chest tube was inserted immediately after the procedure. One patient had a late pneumothorax on the fourth day after the procedure and was treated with a chest tube. Three patients had prophylactic chest tube placements for anticipated pneumothorax due to severely emphysematous lungs. Two patients had moderate hemothorax, which did not require active treatment. Small hematomas occurred in many patients, which did not require any active treatment and were self-limiting. No other major complications occurred in any of the patients.

## Conclusions

Though the basic principles, access routes and complications are similar to those of any other biopsy procedures performed by interventional radiologists, a few challenges are specific to fiducial placement. A thorough knowledge of fiducial placement principles is necessary. Migration of the fiducials is unpredictable and can cause loss of fiducials. Fiducial embolism, though extremely rare, is unique to this procedure and is particularly dangerous if placed in one of the larger pulmonary veins. Atelectasis of the lung following pneumothorax/hemothorax is a limitation while attempting peripheral placement of fiducials. When the bowel is to be traversed, systemic antibiotic treatment may be necessary. Informing the patient about the procedure and being prepared for possible complications is important.

## References

[1] Adler JR, Chang SD, Murphy MJ, Doty J, Geis P, Hancock SL (1997). The Cyberknife: a frameless robotic system for radiosurgery. Stereotact Funct Neurosurg.

[2] Kuo JS, Yu C, Petrovich Z, Apuzzo ML (2003). The CyberKnife stereotactic radiosurgery system: description, installation, and an initial evaluation of use and functionality. Neurosurgery.

[3] Quinn AM (2002). CyberKnife: a robotic radiosurgery system. Clin J Oncol Nurs.

[4] Casamassima F, Cavedon C, Francescon P, Stancanello J, Avanzo M, Cora S (2006). Use of motion tracking in stereotactic body radiotherapy: evaluation of uncertainty in off-target dose distribution and optimization strategies. Acta Oncol.

[5] Yu C, Main W, Taylor D, Kuduvalli G, Apuzzo ML, Adler JR (2004). An anthropomorphic phantom study of the accuracy of Cyberknife spinal radiosurgery. Neurosurgery.

[6] Chang SD, Murphy M, Geis P, Martin DP, Hancock SL, Doty JR (1998). Clinical experience with image-guided robotic radiosurgery (the Cyberknife) in the treatment of brain and spinal cord tumors. Neurol Med Chir.

[7] Whyte RI, Crownover R, Murphy MJ, Martin DP, Rice TW, DeCamp MM (2003). Stereotactic radiosurgery for lung tumors: preliminary report of a phase I trial. Ann Thorac Surg.

[8] Koong AC, Le QT, Ho A, Fong B, Fisher G, Cho C (2004). Phase I study of stereotactic radiosurgery in patients with locally advanced pancreatic cancer. Int J Radiat Oncol Biol Phys.

[9] King CR, Lehmann J, Adler JR, Hai J (2003). CyberKnife radiotherapy for localized prostate cancer: rationale and technical feasibility. Technol Cancer Res Treat.

[10] Herfarth KK, Debus J, Lohr F, Bahner ML, Rhein B, Fritz P (2001). Stereotactic single-dose radiation therapy of liver tumors: results of a phase I/II trial. J Clin Oncol.

[11] Sotiropoulou E, Stathochristopoulou I, Stathopoulos K, Verigos K, Salvaras N, Thanos L (2010). CT-guided fiducial placement for cyberknife stereotactic radiosurgery: an initial experience. Cardiovasc Intervent Radiol.

